# Retraction: Facile preparation of bithiazole-based material for inkjet printed light-emitting electrochemical cell

**DOI:** 10.1039/d0ra90026e

**Published:** 2020-03-31

**Authors:** Jingpei Huo, Wanying Zou, Yubang Zhang, Weilan Chen

**Affiliations:** Electrochemical Corrosion Institute, College of Materials Science and Energy Engineering, Foshan University Foshan People’s Republic of China johnhome222@163.com

## Abstract

Retraction of ‘Facile preparation of bithiazole-based material for inkjet printed light-emitting electrochemical cell’ by Jingpei Huo *et al.*, *RSC Adv.*, 2019, **9**, 6163–6168.

(1) Xiaohong Hu, Qianjun Deng and Dongchu Chen wish to resign as co-authors on the above article.

The corresponding author, Jingpei Huo, has confirmed that they did not participate in any aspect of the paper, from the experimental research to the preparation and submission of the manuscript. The manuscript was prepared and submitted without the knowledge or agreement of these three authors. In addition, they did not give permission for their names to be included as authors on the paper.

The corrected authorship list and affiliations for this paper is as follows:

Jingpei Huo,*^a^ Wanying Zou,^a^ Yubang Zhang,^a^ and Weilan Chen^a^

^a^Electrochemical Corrosion Institute, College of Materials Science and Energy Engineering, Foshan University, Foshan, People’s Republic of China

E-mail: johnhome222@163.com

(2) The Royal Society of Chemistry, with the agreement of the named authors in the corrected authorship list, hereby wholly retracts this *RSC Advances* article due to unattributed text overlap in the Results, Discussion and Conclusion sections of this article and a number of articles published by different authors.^1–7^ With the exception of ref. 6, the articles are not cited in this *RSC Advances* article.

In addition to the text overlap, there is unattributed data overlap with ref. 1 and ref. 5, and concerning inconsistencies have been identified which affect the reliability of the figures. The authors were not able to satisfactorily address these concerns or provide raw data for the figures in the published article. The Royal Society of Chemistry consulted with an independent expert who agreed that, given these significant discrepancies, together with the unattributed text and data overlap, the findings presented in this published article are no longer reliable.

Signed: Jingpei Huo, Wanying Zou, Yubang Zhang, and Weilan Chen

Date: 6^th^ March 2020

Retraction endorsed by Laura Fisher, Executive Editor, *RSC Advances*

## Supplementary Material
